# Development of a Metabolite Sensor for High-Throughput Detection of Aldehydes in *Escherichia Coli*

**DOI:** 10.3389/fbioe.2018.00118

**Published:** 2018-08-23

**Authors:** Cláudio R. Frazão, Victor Maton, Jean M. François, Thomas Walther

**Affiliations:** ^1^LISBP, CNRS, INRA, INSA, Université de Toulouse, Toulouse, France; ^2^Toulouse White Biotechnology (TWB), Toulouse, France

**Keywords:** aldehyde metabolite sensor, YqhC, transcription factor, directed evolution, flow cytometry

## Abstract

We have developed a fluorescence-based metabolite sensor enabling *in vivo* detection of various aldehydes of biotechnological interest in *Escherichia coli*. YqhC is a transcriptional regulator that is known to be involved in the upregulation of the *yqhD-dgkA* operon in the presence of aldehydes. We took advantage of this property by constructing a bi-modular biosensor, in which a sensing module constitutively expresses *yqhC* while a reporter module drives the expression of the *syfp2* reporter gene that is put under control of the *yqhD* promoter. The sensitivity of the sensor has been optimized by engineering the 5′-UTRs of both the sensing and the reporter modules resulting in a 70-fold gain of fluorescence in response to the model compound glycolaldehyde at 5 mM. The optimized sensor further responded to other aldehydes when supplemented to the cultivation medium at concentrations of 1–10 mM. We furthermore showed that this metabolite sensor was functional *in vivo* as it responded to the presence of glycoladehyde that is specifically produced upon induction of a synthetic xylulose-1-phosphate pathway expressed in *E. coli*. This bi-modular sensor can therefore be employed as an exquisite tool for FACS-based ultra-high-throughput screening of aldehyde (over) producing enzymes.

## Introduction

Aldehydes are a class of chemicals with a wide range of applications, namely in the synthesis of rubbers, plastics and formulation of flavors and fragrances. Formaldehyde, butanal and isobutyraldehyde are among the aldehydes produced in quantities >1 Mt per year, while the aromatic aldehydes vanillin and benzaldehyde are the two most widely used flavoring agents in food products (Kunjapur et al., 2014; Rodriguez and Atsumi, 2014). Producing these compounds from inexpensive sugar feedstocks using microbial species is therefore an alternative to the currently established chemical synthesis and plant-extraction processes (Kunjapur and Prather, 2015). Beyond their use as end-products, many aldehydes are intermediates of metabolic pathways resulting in multiple and diversified products (e.g., fatty acids, alcohols, alkanes, carboxylic acids), including those developed by our group leading to the production of glycolic acid (via glycolaldehyde), 1,3-propanediol (via 3-hydroxypropanal), and 2,4-dihydroxybutyrate (via malate semialdehyde) (Akhtar et al., 2013; Walther and Francois, 2014; Cam et al., 2016; Walther et al., 2017). Optimizing those enzymatic activities that produce aldehydes is an essential task during the improvement of the above mentioned synthetic metabolic pathways. However, the rational or evolutionary engineering of such new enzymatic activities can be subject to various bottlenecks. Recent advances in synthetic biology together with decreasing DNA synthesis costs allow today for rapid design and assembly of DNA sequences that may encode individual enzymes or even entire metabolic pathways (Erb et al., 2017; Mori and Shirai, 2018). While an increasingly large sequence space becomes accessible at lower costs and higher rates, the available analytical systems to screen this vast number of sequences for positive variants often do not provide the necessary throughput. Small molecules such as aldehydes can be detected and quantified through the use of conventional chromatography (GC-MS, HPLC-MS) (Berdyshev, 2011) and colorimetric techniques (Schiff's test). However, the analytical throughput of these techniques is far from being exploitable for laboratory evolution campaigns that are based on random mutagenesis. The development of high-throughput screening and selection methods is therefore of crucial importance for successful directed evolution of strains and enzymes aiming at aldehyde overproduction (Cheng et al., 2018).

A variety of devices sensitive to the accumulation of intra- or extracellular small molecules, ions or changes in physical parameters have been evolved using microbial cells (Mahr and Frunzke, 2016). Among those, ligand-responsive transcription factors (TFs) play a major role in cell physiological adaptation. They are DNA-binding proteins that regulate gene expression by physically interacting with specific target molecules (Browning and Busby, 2004; Dietrich et al., 2010). Therefore, they are interesting devices for a broad range of applications, including the construction of whole-cell biosensors or metabolite sensors for detection of, respectively, extracellular or intracellular target molecules (Siedler et al., 2014; Li et al., 2017; Kasey et al., 2018). Metabolite sensors are usually modular. While a sensing module contains a transcriptional regulator which is activated in the presence of a target ligand, a reporter module consisting of a corresponding cognate promoter which drives transcription of a reporter gene (e.g., *lacZ, gfp*, and mutant variants) enables the output of measurable signals (Chong and Ching, 2016). The utilization of metabolite sensors has recently gained particular interest for evolution of bacterial strains (Morgan et al., 2016; Rogers et al., 2016; Liu et al., 2017) and enzymes (Cheng et al., 2015; Kwon et al., 2018).

When metabolite sensors that detect the intracellular production of target molecules are combined with fluorescence-activated cell sorting (FACS) systems, ultra-high throughput analyses and sorting of individual cells become feasible at rates higher than 10^7^ cells screened per hour (Schallmey et al., 2014). While these numbers make metabolite sensors a highly attractive tool for strain and enzyme engineering, the use of these sensors and respective implementation in a screening protocol is still far from being an “off-the-shelf technology” thereby requiring significant research efforts for both, the optimization of the sensor and the screening protocols. In this work, we report on the development of a metabolite sensor for detection of various aldehydes in *E. coli* which employs the aldehyde-responsive transcription factor YqhC to drive the expression of the yellow fluorescent reporter protein SYFP2. Based on 5′-UTR engineering of the sensor and reporter modules, the gain of the fluorescence signal in response to the model compound glycolaldehyde was strongly increased. The best sensor variant detected various extracellularly added aldehydes at concentrations in the range of 1–10 mM. In addition, intracellular production of glycolaldehyde through the synthetic xylulose-1-phosphate pathway was reliably detected. This result showed that the metabolite sensor can be applied in screening systems that rely on the detection of intracellular production of a target aldehyde in live cells.

## Materials and methods

### Chemicals and reagents

All chemicals and solvents were purchased from Sigma-Aldrich unless otherwise stated. Restriction endonucleases and DNA-modifying enzymes were purchased from New England Biolabs and used according to instructions of the manufacturer. DNA plasmid isolation was performed using GeneJET Plasmid Miniprep Kit (Thermo Scientific). DNA extraction from agarose gel was carried out using the GeneJET Gel Extraction Kit (Thermo Scientific). DNA sequencing was carried out by Eurofins SAS (Ebersberg, Germany).

### Plasmid construction

All plasmids and primers used in this study are listed in Tables 1, 2, respectively.

**Table 1:** Strains and plasmids used in this study.

**Name**	**Description**	**Source**
**Strains**	**Genotype**	
DH5α	*e. coli fhuA2 Δ(argF-lacZ)U169 phoA glnV44 Φ80Δ (lacZ)M15 gyrA96 recA1 relA1 endA1 thi-1 hsdR17*	NEB
MG1655	F^−^λ^−^ ilvG- rfb-50 rph-1	ATCC 47076
CF30	MG1655 Δ*sad* Δ*yqhD*	This work
Pen155	MG1655 Δ*xylB*	Cam et al., 2016
CF272	Pen155 Δ*yqhD*	This work
**Plasmids**	**Relevant characteristics**	
pCP20	Amp^R^, temperature-sensitive replicon, expressing FLP recombinase	Cherepanov and Wackernagel, 1995
pZE13	Amp^R^; colE1 ori; promoter P_A1_lacO1	Expressys
pZS23	Kan^R^; pSC101 ori; promoter P_A1_lacO1	Expressys
pZA33	Chm^R^; p15A ori; promoter P_A1_lacO1	Expressys
pEXT20-khkC-aldoB	AmpR; colE1 ori; promoter pTAC: *khkC*: *aldoB*	Cam et al., 2016
pSENS-13	pZS23 derivative; promoter BBa_J23106: RBS_weak_: *yqhC*	This work
pSENS-16	pZS23 derivative; promoter BBa_J23114: RBS_weak_: *yqhC*	This work
pSENS-17	pZS23 derivative; promoter BBa_J23113: RBS_weak_: *yqhC*	This work
pSENS-18	pZS23 derivative; promoter proD: RBS_weak_: *yqhC*	This work
pSENS-19	pZS23 derivative; promoter BBa_J23113: RBS_veryweak_: *yqhC*	This work
pSENS-20	pZS23 derivative; promoter BBa_J23113: RBS_medium_: *yqhC*	This work
pSENS-21	pZS23 derivative; promoter BBa_J23113: RBS_strong_: *yqhC*	This work
pREP-14	pZE13 derivative; promoter P_yqhD_: RBS_01_: *syfp2*	This work
pREP-15	pZE13 derivative; promoter P_yqhD_ extended: RBS_01_: *syfp2*	This work
pREP-22	pZE13 derivative; promoter P_yqhD_ hybrid: RBS_01_: *syfp2*	This work
pZA33-khkC-aldoB	pZA33 derivative; promoter P_A1_lacO1: *khkC*: *aldoB*	This work

**Table 2:** Primers used in this study for plasmid construction and strain validation.

**Primer**	**Sequence (5′-3′)**
**CONSTRUCTION OF SENSING MODULES (pSENS)**	
CF149	tatata*Gtcgag*tttacggctagctcagtcctaggtatagtgctagcGGTCCACCGCTTACCCCCCCAAGGGACGAATAAA**atg**CTACAAAATTGCGCACA
CF154	tgctta*ggatcC*ttaATTCCCCTGCATCG
CF161	taagca*ctcgag*tttatggctagctcagtcctaggtacaatgctagcGGTCCACCGCTTACCCCCCCAAGGGACGAATAAA**atg**CTACAAAATTGCGCACA
CF162	taagca*ctcgag*ctgatggctagctcagtcctagggattatgctagcGGTCCACCGCTTACCCCCCCAAGGGACGAATAAA**atg**CTACAAAATTGCGCACA
CF163	taagca*ctcgag*CACAGCTAACACCACGTC
CF164	TGTGCGCAATTTTGTAGcatTTTATTCGTCCCTTGGGGGGGTAAGCGGTGGACCAAAGTTAAACAAAATTATTTGTAGAGG
CF257	taagca*ctcgag*tttatggctagctcagtcctaggtacaatgctagc**GTCTTAACAAAGGAAAAAATTTACT**atgCTACAAAATTGCGCACA
CF258	taagca*ctcgag*tttatggctagctcagtcctaggtacaatgctagc**AAATTTACTTATAAAGGAGGAGATAG**atgCTACAAAATTGCGCACA
CF259	taagca*ctcgag*tttatggctagctcagtcctaggtacaatgctagc**TCGGAAGAAGAATCGAGGAGGAGGTATCA**atgCTACAAAATTGCGCACA
**CONSTRUCTION OF REPORTER MODULES (pREP)**	
CF155	gaggccctttcgtcttcacctcgagttaCACATCGGGCAACAGTCC
CF156	gtatttaagttggaaagcttAGGGCAGAGAACGATCTG
CF157	tctctgccctaagctttccaacttaaatacaaggaaaataaggaggtcaacATGGTTAGCAAGGGCGAAG
CF158	gtacgcgtaccatgggatccTTATTATTTATACAGCTCATCCATACCC
CF159	TAAGCActcgagttaCACATCGGGCAACAGTC
CF160	tgcttaAAGCTTTTAAACTTGATCGAGAACGCC
CF324	tgcttaAAGCTTGGTCAGTGCGTCCTGCTGATGTGCTCAGTATCATCGCCAGCGCCCTG
**CONSTRUCTION OF ALDEHYDE-SYNTHETIC PATHWAY**	
Pen268	cggctgctaacaaagcccg
Pen269	*gaattc*tgtgtgaaattgttatccgc
Pen321	***tttcacacagaattc*****GTTTAACTTTAA******GAAGGAGATATACC******ATG**GAAGAGAAGCAGATCCTGTGC
Pen322	***ctttgttagcagccg*****ggatcctca**TTAATACGTGTAACAGGCCGTAAACAGA
**STRAIN VERIFICATION**	
Δsad-ver-fw	CTGCCAGCTTCGGCAA
Δsad-ver-rv	GGGTAAAGTCGCGGATTAT
ΔyqhD-ver-fw (Pen15)	CAAGCGGCAAATCTCTTCAC
ΔyqhD-ver-rv (CF346)	TGGATTAGCCATACGTTCCT

#### Construction of sensing modules

The upstream regions (including variable strength constitutive promoters and ribosome binding sites) were introduced into the forward primer that together with the reverse primer CF154 served to amplify the wild-type *yqhC* gene from genomic DNA of *E. coli* K-12 *substr*. MG1655 (ATCC 47076). Additionally, the primers used allowed the insertion of unique restriction sites upstream and downstream of the amplified fragments. Resulting PCR products and the low-copy vector backbone pZS23 (Expressys, Germany) were digested with *Xho*I and *Bam*HI restriction enzymes, gel purified and ligated with T4 DNA ligase. The resulting plasmids were transformed into DH5α competent *E. coli* cells (New England Biolabs) and inserts verified by DNA sequencing.

#### Construction of reporter modules

The YqhD promoter region was PCR amplified from genomic DNA with primers pairs CF155/CF156, while the *syfp2* gene (synthesized by Eurofins) was amplified with primers CF157/CF158. DNA fragments were gel purified and assembled by homologous recombination with the *Xho*I/*Bam*HI-digested pZE13 vector using the NEBuilder® HiFi DNA Assembly kit (New England Biolabs). The resulting plasmid was named pREP-14. For construction of pREP-15 and pREP-22 vectors, promoter regions of *yqhD* gene were PCR-amplified from *E. coli* genomic DNA using respectively the primer pairs CF159/160 and CF155/324. The obtained PCR products and pREP-14 plasmid were digested (*Xho*I, *Hind*III), gel-purified and complementary-ends ligated. After sequencing analysis, the resulting plasmids were named pREP-15 and pREP-22, respectively.

#### Construction of pZA33-khkC-aldoB

The khkC-aldoB operon was amplified by PCR from pEXT20-khkC-aldoB (Cam et al., 2016) using the primer pairs Pen268/Pen269, while the medium-copy vector pZA33 (Expressys, Germany) was PCR-linearized using primer pairs Pen321/Pen322. Resulting PCR products were gel purified and assembled by homologous recombination using the NEBuilder® HiFi DNA Assembly kit (New England Biolabs). The resulting plasmid was transformed into DH5α competent *E. coli* cells and the assembled operon verified by DNA sequencing.

### Strain construction and growth conditions

*Escherichia coli* K-12 substr. MG1655 (ATCC 47076) was used as the parental strain for all constructions in this study. Deletion of *yqhD* and *sad* genes was achieved using the phage transduction method adapted from Miller (1992). The phage lysate was prepared from strains of the KEIO (Baba et al., 2006) collection which carried single deletions. Positive clones were selected on LB agar plates containing kanamycin (50 μg mL^−1^) and verified by PCR analysis. The kan cassette was removed from the genome by expressing FLP recombinase from the pCP20 plasmid (Cherepanov and Wackernagel, 1995) and correct excision of the cassette was verified by PCR using locus specific primers (Table 2). Plasmids were transformed into the target *E. coli* strains using standard protocols (Sambrook et al., 1989).

The cultures of *E. coli* strains were carried out at 37°C on a rotary shaker running at 200 rpm in a M9 mineral medium which, unless otherwise stated, contained per liter: 20 g glucose, 18 g Na_2_${\text{HPO}}_{4}^{*}$12H_2_O, 3 g KH_2_PO_4_, 0.5 g NaCl, 2 g NH_4_Cl, 0.5 g ${\text{MgSO}}_{4}^{*}$7H_2_O, 0.015 ${\text{CaCl}}_{2}^{*}$2H_2_O, 1 ml of 0.06 M FeCl_3_ stock solution prepared in 100 times diluted concentrated HCl, 2 ml of 10 mM thiamine HCl stock solution, 20 g MOPS, and 1 ml of trace element solution (containing per liter: 0.04 g Na_2_EDTA^*^2H2O, 0.18 g ${\text{CoCl}}_{2}^{*}$6H_2_O, ${\text{ZnSO}}_{4}^{*}$7H_2_O, 0.04 g Na_2_${\text{MoO}}_{4}^{*}$2H_2_O, 0.01 g H_3_BO_3_, 0.12 g ${\text{MnSO}}_{4}^{*}$H2O, 0.12 g ${\text{CuCl}}_{2}^{*}$H_2_O). The pH was adjusted to 7 and the medium was filter-sterilized. The antibiotics ampicillin and kanamycin sulfate were added when required at concentrations of 100 and 50 mg L^−1^, respectively.

For assaying *in vivo* production of glycolaldehyde by the *E. coli* strain CF272 harboring the xylulose-1-phosphate pathway and metabolite sensor, the experiments were carried out as follows. Pre-cultures were grown in 10 mL M9 mineral medium in the presence of the antibiotics ampicillin, kanamycin sulfate and chloramphenicol at 100, 50, and 35 mg mL^−1^. After an overnight incubation, cells were spun down by centrifugation (4000 rpm, 10 min at 4°C) and washed with sterile water. They were resuspended at an initial OD_600_ of 0.5 in 25 mL of fresh M9 mineral medium containing 1 mM IPTG and appropriate antibiotics and in the presence of glucose or a mixture of glucose/xylose. Cell growth was followed by monitoring OD_600_, and samples were regularly withdrawn for flow cytometry analyses and sugars consumption.

### Microtiter plate screening system

#### Screening of biosensor-strains

Pre-cultures were grown overnight in 5 mL of M9 mineral medium (37°C, 200 rpm). They were used to inoculate 10 mL of M9 mineral medium supplemented with the appropriate antibiotics starting at an initial OD_600_ of 0.2 in 50 mL falcon tube flasks placed on a rotary shaker set at 200 rpm and at 37°C. When OD_600_ reached ~0.6, 200 μL of cell culture were transferred in a 96-well plate and supplemented with glycolaldehyde at a final concentration of 5 mM. During the induction phase, microplates were incubated at 37°C with an orbital frequency at 807 rpm (Epoch 2, BioTek). After 4 h of incubation, single-cell fluorescence was measured by flow cytometry.

#### Aldehyde dose-response curves

Pre-cultures were grown overnight in 5 mL of M9 mineral medium (37°C, 200 rpm). The biomass needed to start main cultures with a starting OD_600_ of 0.2 was transferred to 250 non-baffled shake flasks containing 25 mL of M9 mineral medium with appropriate antibiotics (37°C, 200 rpm). When OD_600_ reached ~0.6, 200 μL of cell culture were inoculated in a 96-well plate and supplemented with aldehydes at the desired concentrations. During the induction phase, microplates were incubated at 37°C with an orbital frequency at 807 rpm (Epoch 2, BioTek). After 12 h of incubation, single-cell fluorescence was measured by flow cytometry.

### Flow cytometry

Flow cytometry measurements were performed with an Attune™ Acoustic Flow Cytometer (Life Technologies) with 488 nm excitation. Forward-scatter characteristics (FSC) and side-scatter characteristics (SSC) were detected as small-angle and large-angle scatters of the 488 nm laser, respectively. SYFP2 fluorescence was detected using a 530/30 nm (channel BL1) band-pass filter set. Data were analyzed using the Attune™ software (Life Technologies). A total of 100,000 events was recorded per sample, and electronic gating was applied on the densest subset of cells on the basis of forward- vs. side-scatter height. The same gate was used to estimate geometric mean levels of SYFP2 fluorescence.

### Analytical methods

The concentrations of glucose, xylose and glycolaldehyde were determined on a Dionex Ultimate 3,000 HPLC system (Thermo Scientific, France) equipped with a RI detector (RID-10A, Shimadzu, Japan). The sample injection volume was 20 μL, and the compounds were separated in an Aminex HPX-87H column protected by a Micro-Guard Cation H pre-column (BioRad, USA). The separation was performed at 35°C with 1.25 mM H_2_SO_4_ at 0.5 mL min^−1^ as mobile phase. All samples were centrifuged (2 min at 13,000 rpm) and syringe-filtered (0.2 μm), and the resulting supernatant kept at −20°C until analysis.

### Statistical methods

All statistical analyses were conducted in Microsoft Excel® using the Analysis ToolPak package. A two-tailed unpaired *t*-test was to use to compare fluorescence induction levels, in which an alpha level of *p* < 0.05 was set for significance.

## Results

### YqhC can be employed as an aldehyde sensor

**Figure 1:**
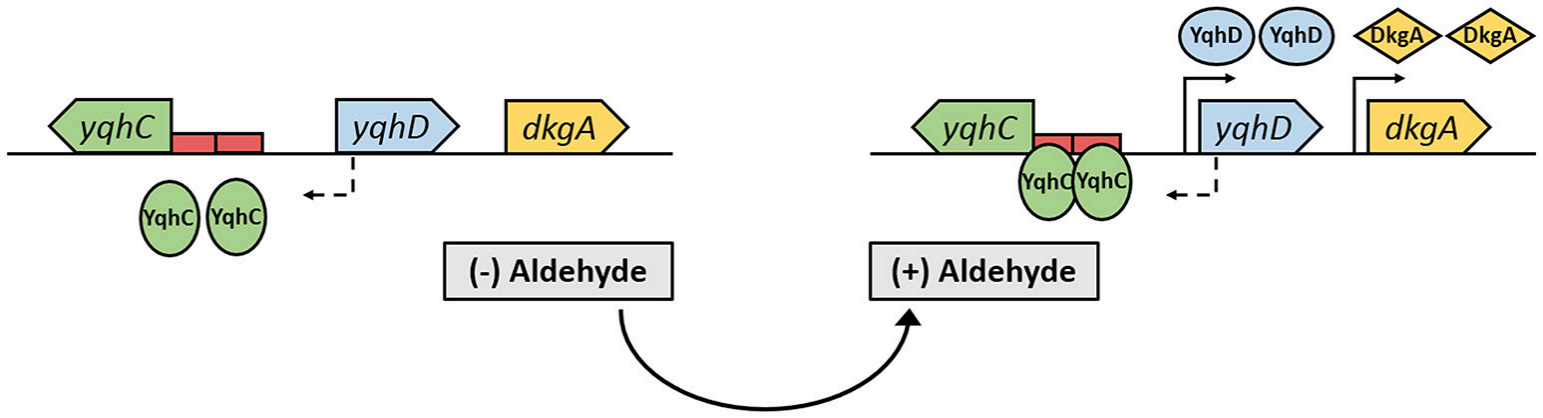
The *yqhC*/*D-dkgA* operon in *E. coli* MG1655 (Keseler et al., 2017). When cells are exposed to aldehydes, the constitutively expressed transcription factor YqhC binds to the promoter region of *yqhD* that contains a SoxS-like binding sequence as well as a 24-bp palindrome (red rectangles), enhancing/activating transcription of *yqhD* and *dkgA* genes, resulting in the increased expression of the NADPH-dependent aldehyde reductases YqhD and DkgA. Solid and dashed arrows represent the transcription start site of divergently transcribed genes.

In a previous study, we identified YqhD as the major glycolaldehyde reductase in *E. coli* (Cam et al., 2016), while others demonstrated this enzyme to be active on a broad range of short-chain aldehydes (e.g., butyraldehyde, 3-hydroypropanal, acrolein) (Jarboe, 2011). In a genomic context, YqhD is expressed from the *yqhD*-*dkgA* operon that is known to be induced by the divergently transcribed transcriptional regulator YqhC (Figure 1) (Lee et al., 2010; Turner et al., 2011). Genome-wide transcriptome studies from our group, furthermore, revealed *yqhD* and *dkgA* genes to be strongly up-regulated (by 26- and 10-fold, respectively) when wild-type *E. coli* cells were exposed to 10 mM glycolaldehyde (Cam et al., 2016). On the basis of these observations, we hypothesized that the transcription factor YqhC may be used to engineer an *in vivo* aldehyde-sensor system in *E. coli*.

**Figure 2:**
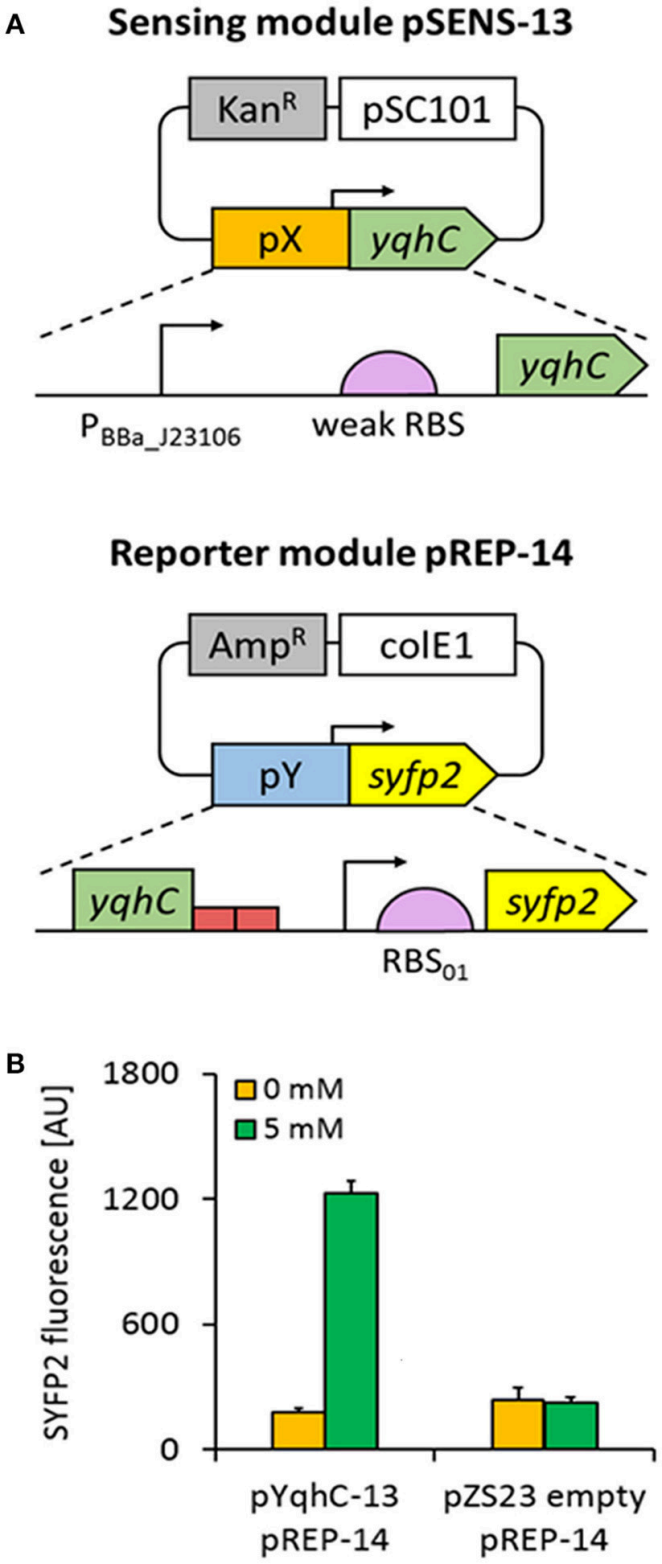
Design of the YqhC-based aldehyde sensor. **(A)** The sensing module pSENS-13 consists of a low-copy plasmid in which *yqhC* is under control of a medium-strength constitutive promoter (P_BBa_J23106_) and a weak ribosome binding site. The reporter module pREP-14 was built by fusing the *syfp2* reporter gene (preceded by a strong RBS) to the YqhC cognate promoter in a high-copy vector. The 5′-UTR regions containing regulatory elements responsible by transcription of *yqhC* and reporter genes were named pX and pY, respectively. In each module, the antibiotic resistance marker and origin of replication is shown (gray and white boxes, respectively). **(B)** Fluorescence variation upon aldehyde exposure of engineered *E. coli* strain co-transformed with pSENS-13 and pREP-14. In a control experiment, the host strain was transformed with pZS23 and pREP-14. All strains are derived from the host strain CF30 (*E. coli* MG1655 Δ*sad* Δ*yqhD*). Cells were cultivated in M9 mineral medium containing 20 g L^−1^ glucose and incubated for 4 h with 5 mM glycolaldehyde when OD_600_ reached ~0.6. SYFP2 fluorescence was calculated using cytometry data based on geometric mean. The reported values represent the mean ± S.D (*n* ≥ 2).

To investigate this possibility, we constructed a bi-modular system where the sensing module (named pSENS-13) drove constitutive expression of the regulatory protein YqhC from a low-copy number vector. In the reporter module (named pREP-14), expression of the super yellow fluorescent protein SYFP2 (Kremers et al., 2006) was placed under transcriptional control of the *yqhD* promoter region, that included 150-nt of the *yqhC* coding sequence and the adjacent 108-nt *yqhC/D* intergenic region (until 28-nt downstream the transcription start site where the putative ribosome binding site pre-sequence upstream *yqhD* gene starts) (Figure 2A). To maximize protein expression, a 35-nt strong ribosome binding site (RBS_01) was designed using the RBS calculator tool (Salis et al., 2009) and placed in front of the *syfp2* reporter gene (Table 3 shows DNA sequence). Reporter and sensing modules were then co-transformed into the parent strain CF30 (MG1655 Δ*sad* Δ*yqhD*), that was chosen as the host for evaluating biosensor-strains. Deletion of the *yqhD* and *sad* genes was expected to minimize intracellular degradation of short-chain aldehydes and semialdehydes, respectively. All resulting biosensor-strains were cultivated in M9 mineral medium and candidate aldehydes were added to the exponentially growing cells when OD_600_ reached ~0.6. It is of note that the utilization of a minimal medium resulted in a strong reduction of cell background fluorescence, as opposed to utilization of Luria Broth (LB) and 2x Yeast tryptone (YT) rich media (data not shown). Since we had previously found that glycolaldehyde was a potent inducer of the YqhC-dependent transcriptional response (Cam et al., 2016), we first investigated the behavior of our sensor in response to this compound at a non-lethal concentration of 5 mM. After a 4 h incubation period, fluorescence intensity was measured at the single-cell level by flow cytometry and found to be increased by 6.8-fold when compared to cells which were cultivated in the absence of this aldehyde (Figure 2B). In a control experiment, we co-transformed the host strain CF30 with the pZS23 empty plasmid and the reporter module pREP-14. The resulting strain displayed no fluorescence increase upon aldehyde exposure, thereby confirming the feasibility of the developed metabolite sensor.

**Table 3:** Constitutive promoters and RBS used for construction of sensing modules in the metabolite sensor.

**Reference**	**Sequence (5′-3′)**	**Strength**
**PROMOTERS**		
BBa_J23106^a^	TTTACGGCTAGCTCAGTCCTAGGTATAGTGCTAGC	1,185 a.u.^a^
BBa_J23114^a^	TTTATGGCTAGCTCAGTCCTAGGTACAATGCTAGC	256 a.u.^a^
BBa_J23113^a^	CTGATGGCTAGCTCAGTCCTAGGGATTATGCTAGC	21 a.u.^a^
proD, insulated	CACAGCTAACACCACGTCGTCCCTATCTGCTGCCCTAGGTCT ATGAGTGGTTGCTGGATAACTTTACGGGCATGCATAAGGCTCGTATAATATATT CAGGGAGACCACAACGGTTTCCCTCTACAAATAATT TTGTTTAACTTT	5,191. a.u^b^
**RBS**^c^		
Weak	GGTCCACCGCTTACCCCCCCAAGGGACGAATAAA	10,000 T.I.R.
Very weak	GTCTTAACAAAGGAAAAAATTTACT	1,000 T.I.R.
Medium	AAATTTACTTATAAAGGAGGAGATAG	100,000 T.I.R.
Strong	TCGGAAGAAGAATCGAGGAGGAGGTATCA	1,000,000 T.I.R.

a *Nomenclature of promoters and relative promoter strengths as in Registry of Standard Biological Parts (http://parts.igem.org/Part:BBa_J23114)*.

b *Strength calculated based on information available at Registry of Standard Biological Parts (http://parts.igem.org/Part:BBa_J23114) and study from Davis and co-workers (Davis et al., 2011)*.

c *RBS with various strengths were designed using online RBS calculator tool (https://salislab.net/software/forward)*.

### Engineering biosensor modules for improved aldehyde detection

We next set out to evaluate the effect of varying *yqhC* expression levels on the strength of the fluorescence signal by engineering promoter and RBS sequences in the sensing module (Table 4). We first tested the effect of three alternative constitutive promoters with different characteristics (strength, insulation) immediately upstream of the weak RBS that controlled protein expression from the *yqhC* coding sequence (see DNA sequences in Table 3). Whilst keeping reporter module pREP-14 unaltered in the host strain, co-transformation with the engineered pSENS-X plasmid variants (in which X = 16–18) resulted in distinct behaviors regarding fluorescence induction ratios upon glycolaldehyde exposure (Table 4). The expression of *yqhC* gene under control of a weak constitutive promoter (P_BBa_J23114_) resulted in the highest fluorescence induction ratios (18.9-fold, *p* < 0.001), and we therefore used the corresponding plasmid pSENS-16 as the backbone for further modifications. The implementation of ribosome binding sites of variable strengths (pSENS-X, in which X = 19–21) resulted in further improved fluorescence induction ratios of up to 32.6-fold when the pSENS-20 plasmid (medium-strength RBS, *p* < 0.01) was used.

**Table 4:** Fluorescence induction upon aldehyde exposure of engineered *E. coli* strains co-transformed with various sensing modules and pREP-14 as reporter module.

**Sensing module**			**Reporter module**	**Fluorescence induction**^b^	
**Name**	**Promoter strength**	**RBS strength**^a^		
pSENS-13	Medium (P_BBa_J23106_)	Weak	pREP-14	6.8 (±1.0)
**PROMOTER ENGINEERING**				
pSENS-16	Weak (P_BBa_J23114_)	Weak	pREP-14	18.9 (±1.1)^***^
pSENS-17	Very weak (P_BBa_J23113_)	Weak	pREP-14	4.6 (±0.5)
pSENS-18	Strong (proD, insulated)	Weak	pREP-14	13.0 (±1.9)
**RBS Engineering**				
pSENS-19	Weak (P_BBa_J23114_)	Very weak	pREP-14	28.2 (±0.4)
pSENS-20	Weak (P_BBa_J23114_)	Medium	pREP-14	32.6 (±1.6)^**^
pSENS-21	Weak (P_BBa_J23114_)	Strong	pREP-14	24.6 (±6.9)

*All strains are derived from the host strain CF30 (E. coli MG1655 Δsad ΔyqhD). Cells were cultivated in M9 mineral medium containing 20 g L^−1^ glucose and incubated for 4 h with 5 mM glycolaldehyde when OD_600_ reached ~0.6. SYFP2 fluorescence was calculated using cytometry data based on geometric mean. The reported values represent the mean ± S.D. (n ≥ 2)*.

a *Strengths of RBS driving yqhC expression were determined using the RBS calculator tool and vary across 3-orders of magnitude*.

b *Ratio between single-fluorescence values of aldehyde-induced and uninduced cells*.

*** p < 0.001, pSENS16 significant when compared to sensing modules with weak RBS strength (pSENS13,17,18). For comparison between 16 and group with weak RBS strengths

** *p < 0.01, pSENS20 significant when compared to sensing modules with weak promoter strength (pSENS16,19,21)*.

**Figure 3:**
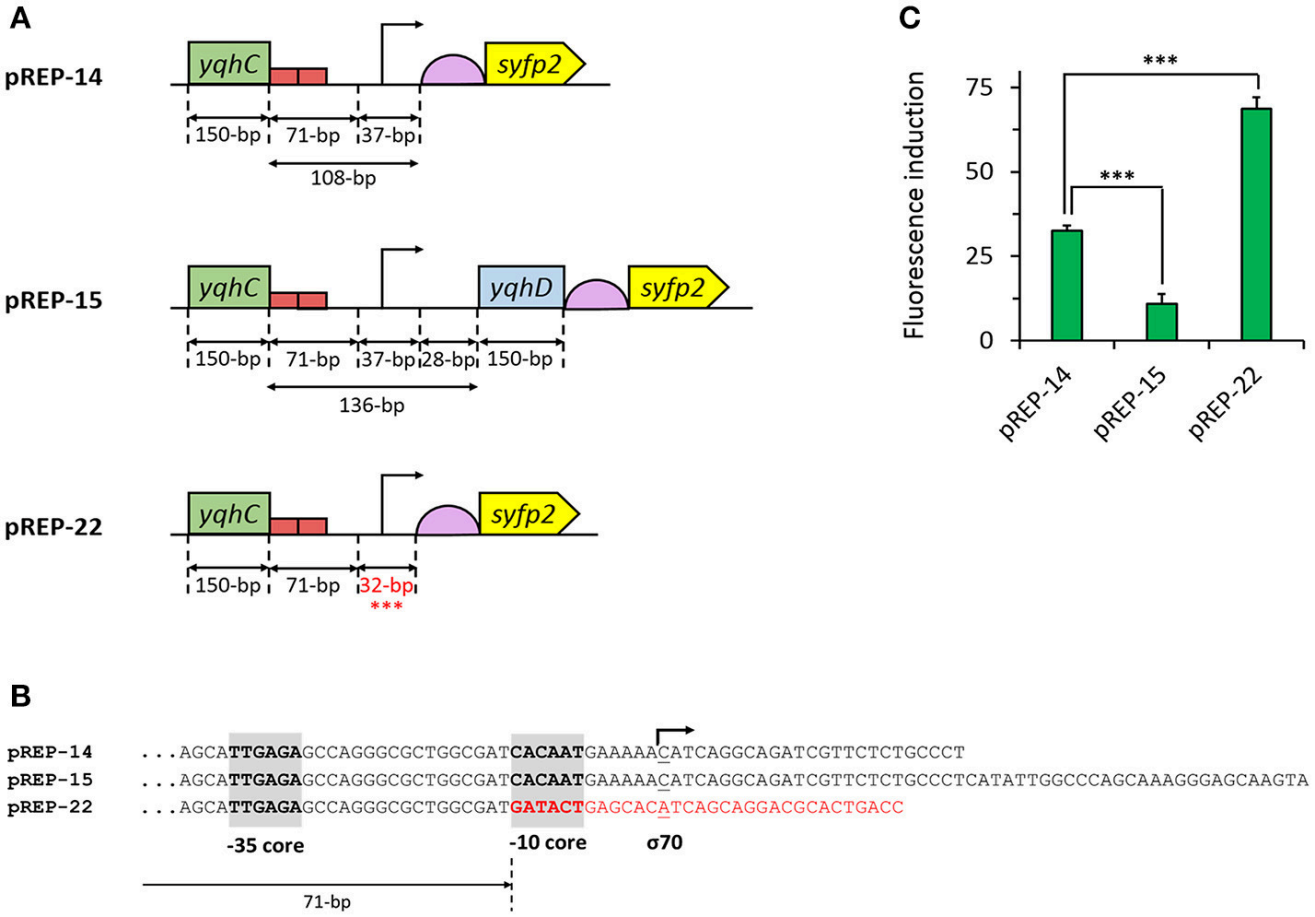
Engineering of the 5′-UTR of the reporter module. **(A)** Schematic map of characteristic sequence elements in the sensor module. **(B)** Comparison of promoter sequences in the pREP-X reporter modules. **(C)** Fluorescence induction upon aldehyde exposure of engineered *E. coli* strains co-transformed with pSENS-13 and various reporter module. All strains are derived from the host strain CF30 (*E. coli* MG1655 Δ*sad* Δ*yqhD*). Cells were cultivated in M9 mineral medium containing 20 g L^−1^ glucose and incubated for 4 h with 5 mM glycolaldehyde when OD_600_ reached ~0.6. SYFP2 fluorescence was calculated using cytometry data based on geometric mean. The reported values represent the mean ± S.D (*n* ≥ 2). ***Indicates that the values are significantly different with a *p* value < 0.05.

Having optimized the sensing module, we next improved the aldehyde sensor by engineering the promoter region of the reporter module pREP-14 (Figure 3). We first created the plasmid pREP-15 by extending the promoter region to include the full *yqhC/D* intergenic region plus the adjacent 150-nt downstream coding region of *yqhD* followed by a stop codon (Figure 3A). The additional introduction of this nucleotidic region was made in an attempt to include possibly missing uncharacterized motifs present in the genome of *E. coli*. However, significantly lower fluorescence induction ratios (10.9-fold) were observed upon aldehyde exposure when compared to pREP-14 (32.6-fold, *p* < 0.001) (Figure 3C). We therefore focused on the optimization of the promoter region in the pREP-14 module to further enhance SYFP2 expression. In the genome of *E. coli*, the *yqhD* gene is preceded by the−35 and−10 elements TTGAGA and CACAAT, respectively, to which RNA polymerase and the sigma factor σ^70^ bind to initiate transcription (with C as the initiation element) (Lee et al., 2010). This configuration was maintained in pREP-14 to control expression of SYFP2. But since the sequence of the−10 promoter core element deviates from the consensus sequence for σ^70^-dependent promoters (-35: TTGACA;−10: TATAAT; initiation element: A) (Hook-Barnard et al., 2006), we decided to replace the−10 core and downstream distal elements in pREP-14 by those found in the well-characterized synthetic IPTG-inducible and σ^70^-dependent promoter P_LlacO−1_ (Figure 3B). When the resulting plasmid pREP-22 was used to detect glycolaldehyde, we observed a 70-fold fluorescence induction ratio (Figure 3C), which corresponded to a 2-fold improvement when compared to the utilization of plasmid pREP-14 as reporter module (*p* < 0.001). Therefore, the plasmids pSENS-20 and pREP-22 were used as sensing and reporter modules for subsequent experiments.

### The YqhC-based aldehyde sensor detects various aldehydes

**Figure 4:**
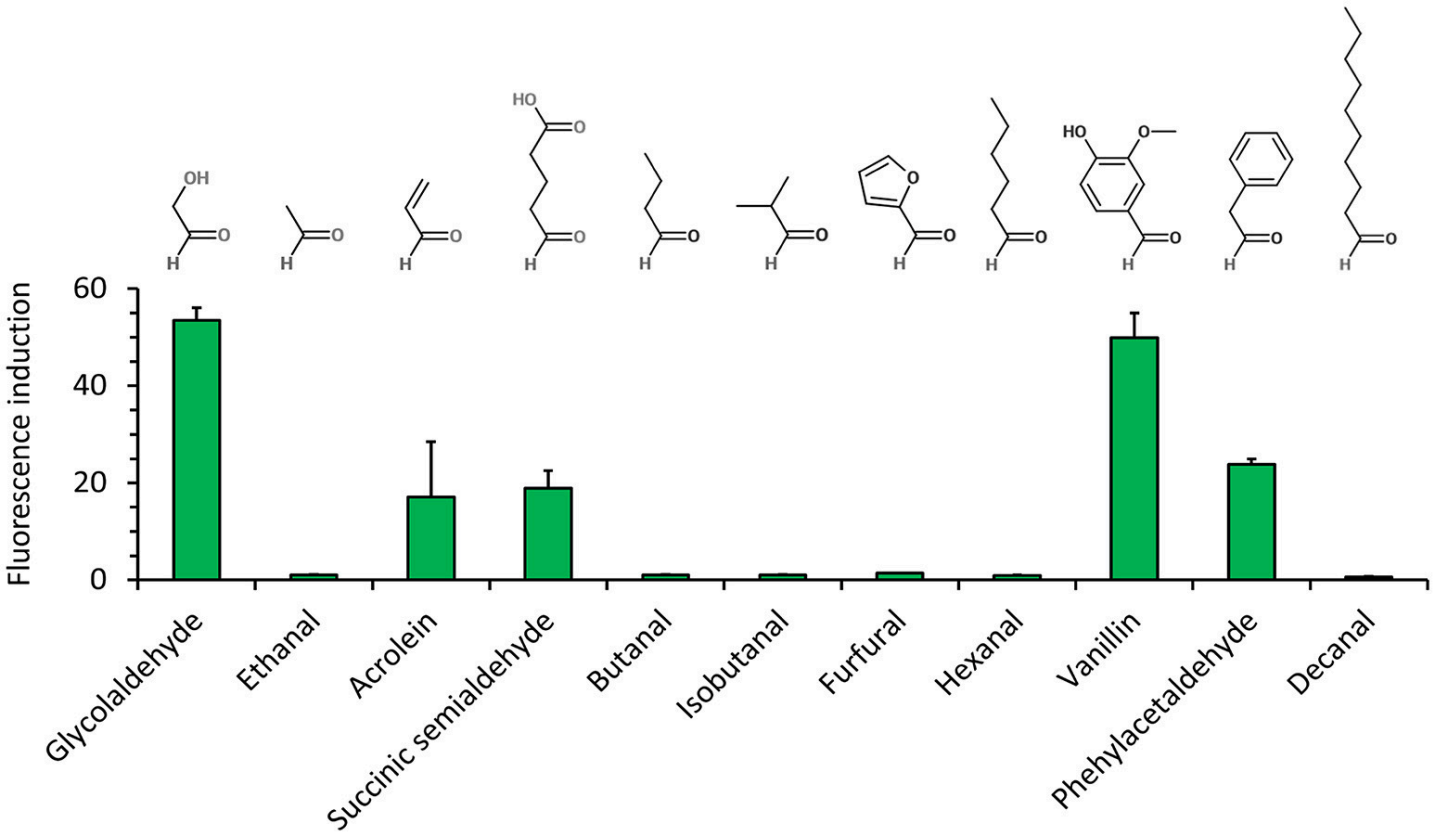
Aldehyde detection spectrum (at a concentration of 5 mM) of *E. coli* host strain CF30 (MG1655 Δ*sad* Δ*yqhD*) harboring pSENS-20 and pREP-22 as sensing and reporter modules, respectivelyCells were cultivated in M9 mineral medium containing 20 g L^−1^ glucose and incubated for 12 h with aldehyde inducer when OD_600_ reached ~0.6. SYFP2 fluorescence was calculated using cytometry data based on geometric mean. The reported values represent the mean ± S.D. (*n* ≥ 2).

**Figure 5:**
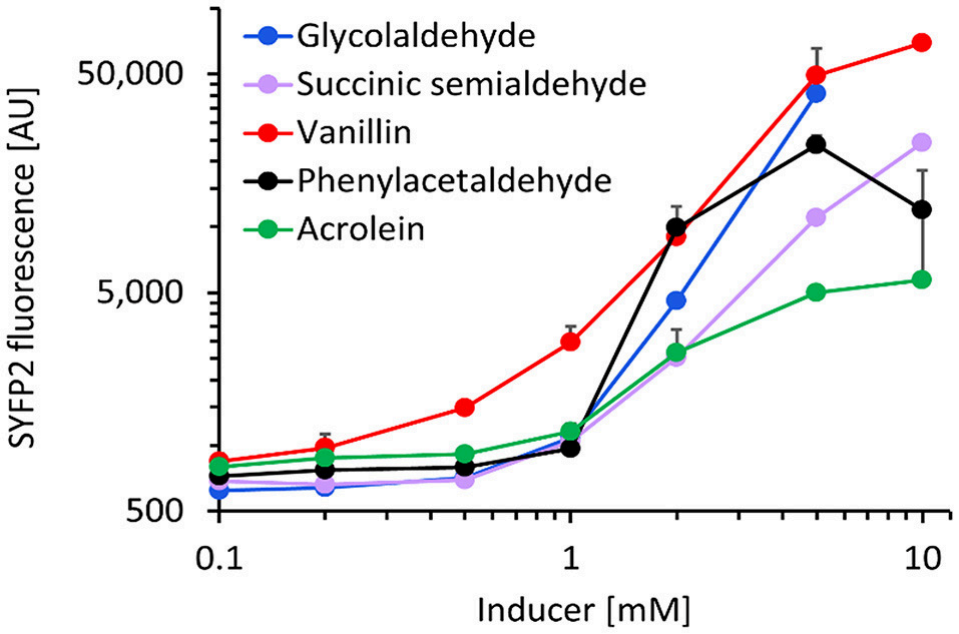
Dose-response curve of *E. coli* host strain CF30 (MG1655 Δ*sad* Δ*yqhD*) harboring pSENS-20 and pREP-22 as sensing and reporter modules, respectively, when exposed to various aldehydes in the concentration range of 0.1–10 mM. Cells were cultivated in M9 mineral medium containing 20 g L^−1^ glucose and incubated for 12 h with aldehyde inducer when OD_600_ reached ~0.6. SYFP2 fluorescence was calculated using cytometry data based on geometric mean. The reported values represent the mean ± S.D. (*n* ≥ 2).

We next characterized the best metabolite sensor in response to the presence of various aldehydes at a concentration of 5 mM (Figure 4). After incubating exponentially-growing cells with candidate aldehydes, we did not observe a clear correlation between the fluorescence induction and the chain length or the chemical structure of the aldehyde, although the presence of a benzyl group in aldehyde molecules (vanillin and phenylacetaldehyde) resulted in comparatively high fluorescence. Additionally, the presence of the short-chain aldehydes acrolein and succinic semialdehyde led to inductions up to 20-fold when compared to non-induced cells, whereas the biosensor was almost insensitive to butanal, furfural, and hexanal. The dose-response curves for the fluorescence-inducing compounds are depicted in Figure 5. With the exception of vanillin, they show that extracellular aldehyde concentrations above 1 mM were necessary to trigger a significant increase of the fluorescence signal.

### The metabolite biosensor can detect *in vivo* production of glycolaldehyde

**Figure 6:**
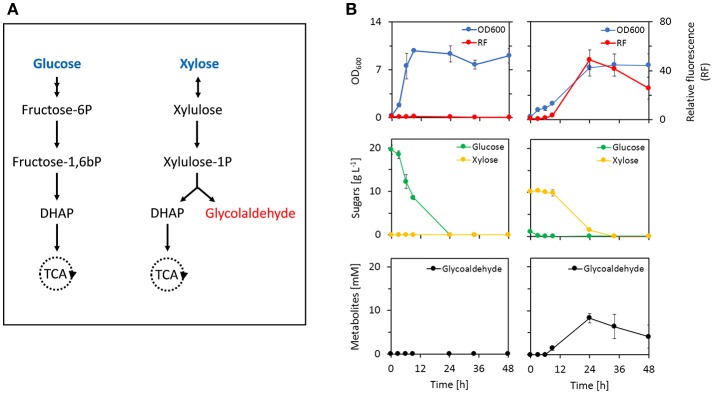
Monitoring of *in vivo* production of glycolaldehyde by an artificial metabolic pathway using the aldehyde metabolite sensor. **(A)** Natural glucose assimilation pathway (top) and synthetic xylulose-1-phosphate pathway (bottom). **(B)** Fermentation profile of *E. coli* host strain CF272 (MG1655 Δ*xylB* Δ*yqhD*) co-transformed with pYqhC-20, pREP-22, and pZA33-khkC-aldoB plasmids in the presence of glucose (left) or a mixture of glucose/xylose (right). Fluorescence induction was calculated from the ratio of SYFP2 fluorescence at a given time point and the SYFP2 fluorescence at the beginning of the cultivation. Fluorescence values were calculated using cytometry data based on geometric mean. The reported values represent the mean ± S.D. (*n* ≥ 2). Glycolaldehyde was determined in the extracellular medium by HPLC coupled to RI detection system. As this compound elutes almost at the same retention time as glycolic acid, and that this later can be detected by UV, a simple visualization of the UV chromatograms confirmed that there was no glycolic acid produced under this condition.

To explore the applicability of the biosensor as a tool for strain and/or enzyme evolution, we monitored intracellular production of glycolaldehyde during induction of a previously developed xylulose-1-phosphate (X1P) synthetic metabolic pathway (Cam et al., 2016). In this pathway (Figure 6A), xylose is first converted into (D)-xylulose by xylose isomerase (XylA, *E. coli*). Xylulose is then phosphorylated by an enzyme with xylulose-1-kinase activity (KhkC, *Homo sapiens*) before the resulting xylulose-1-phosphate is cleaved into glycolaldehyde and dihydroxyacetone phosphate (DHAP) by a xylulose-1-phosphate aldolase (AldoB, *H. sapiens*). While glycolaldehyde can be further metabolized into either ethylene glycol or glycolic acid, DHAP is metabolized through the Embden-Meyerhof-Parnas pathway thereby enabling cell growth. It was previously shown that deletion of the xylulose-5-kinase encoding gene *xylB* was necessary to deviate the xylose-derived carbon flux into the synthetic pathway (Cam et al., 2016). Therefore, we evaluated whether the glycolaldehyde production via the synthetic pathway could be monitored by our metabolite sensor in an *E. coli* Δ*xylB* Δ*yqhD* mutant strain that expresses the aldehyde sensor modules (pSENS-20, pREP-22) and the synthetic pathway (pZA33-khkC-aldoB). Deletion of *yqhD* served to minimize reduction of glycolaldehyde to ethylene glycol and under these conditions, the production of glycolic acid is very low (Cam et al., 2016). The resulting strain was cultivated in mineral medium containing either 20 g L^−1^ glucose or a mixture of 1 g L^−1^ glucose and 10 g L^−1^ xylose as carbon sources. In both cases, expression of the synthetic pathway was induced by the addition of 1 mM IPTG at the start of the culture. Rapid growth was observed during the cultivation on glucose, resulting in a depletion of carbon source in the first 24 h of the cultivation (Figure 6B). Cells growing on glucose exhibited no increase in fluorescence indicating that neither glycolaldehyde nor any other aldehyde was produced at levels that may be detected by our sensor. A different behavior was observed when cells were cultivated on the glucose/xylose mixture. As expected, glucose was first consumed resulting in a doubling of the cell density after the first 3 h of the cultivation. During the diauxic shift which lasted approximately 3 h, cell density increased only very marginally before the growth rate increased again concomitant with utilization of xylose as carbon source. The fluorescence signal further mirrors this behavior in that there was no observable increase of fluorescence during growth on glucose and during the diauxic shift. Only upon induction of the xylose-1-phosphate pathway by xylose, an increase in single-cell fluorescence by more than 40-fold was measured, which nicely correlated with the accumulation of glycolaldehyde in the medium.

## Discussion

The success in improving individual enzymes and/or entire metabolic pathways by directed evolution is often hindered by the lack of high-throughput screening/selection methods enabling detection of target phenotypes (Cheng et al., 2018). Specific detection of small molecules is often required when large mutant libraries are screened for over-producing strains or improved enzyme activities. In the present study, we demonstrate the design and implementation of a metabolite sensor system for the detection of various aldehydes in *E. coli*. While other aldehyde-sensing systems have previously been developed on the basis of invasive and indirect sampling methods (Pariente et al., 1995), our system allows for direct aldehyde detection and may additionally enable FACS-based high-throughput screening and selection for aldehyde-producing enzymes or strains.

In this work, YqhC served as the transcriptional regulator to detect the presence of an aldehyde and to induce expression of a fluorescent marker protein. Turner and colleagues previously constructed a YqhC-based metabolite sensor in *E. coli*, in which the firefly luciferase-encoding gene was under transcriptional control of putative *yqhD* promoter region in a low-copy number plasmid (Turner et al., 2011). Albeit the genomically expressed YqhC levels ensured a light response upon aldehyde exposure, the low measurable output signals observed from cell lysates were not compatible with high-throughput screening purposes, in which cell sorting is desired. In those cases, engineering poor ligand-transcription factor binding through protein or 5′-UTR engineering has previously been reported to dramatically change the response profiles of metabolite sensors (Becskei et al., 2005; Kim et al., 2005). To this end, we therefore constructed an alternative bi-modular aldehyde sensor based on YqhC. A sensing module drives constitutive expression of *yqhC* gene, while a reporter module drives expression of *syfp2* reporter gene under transcriptional control of *yqhD* promoter region. One of the major challenges when developing metabolite sensors is to optimize their sensitivity and dynamic range. We thus used SYFP2 as a reporter protein since it allows for optimized folding, maturation and superior brightness when compared to sfGFP and eYFP autofluorescent protein variants (Kremers et al., 2006). We then fine-tuned the expression levels of YqhC by engineering the promoter and RBS region of the reporter module, resulting in a 10-fold improvement over the initial sensor system. Our results suggest the previously reported concept of two-module metabolite sensors (Xue et al., 2014; Li et al., 2017) to be particularly useful when it comes to optimization tasks, since it enables to independently fine-tune each of its parts.

The specificity and dynamic range responses of our biosensor were evaluated with respect to a spectrum of aldehydes, including short, medium carbon chain and aromatic compounds. At variance to Turner and colleagues who reported a YqhC-dependent transcriptional activation of the firefly-luciferase encoding reporter gene to ethanal, propanal, butanal, methylglyoxal, and lignocellulose inhibitors (furfural, cinnamaldehyde) (Turner et al., 2011), our biosensor was almost insensitive to some of these compounds, but responded to others such as glycolaldehyde, vanillin, and phenylacetaldehyde. The discrepancy between our results and those from Turner and colleagues can be explained in part by the difference in the genomic design of the constructed sensor. In our case, we employed a bi-modular sensor in which the *yqhC* gene is used out of its natural genomic context and expressed constitutively from a low copy plasmid, while the reporter module bears the syfp2 gene preceded by a strong RBS and flanked upstream by a promoter region that included 150-nt of yqhC coding sequence plus 108-nt of adjacent *yqhC/D* intergenic region. On the other side, a different reporter gene (leading to the expression of the firefly luciferase) immediately preceded by the *yqhD* promoter region was constructed by the Ingram group to investigate the response to aldehydes in a *E. coli* strain expressing YqhC from its genomic locus (Turner et al., 2011). In addition and whatsoever the difference between the two sensors, the structural diversity of the aldehydes that exhibit transcriptional induction raised questions about the mechanism by which YqhC may interact with these compounds. While the apparent lack of specificity can be regarded as a disadvantage, we instead consider it as a appropriate feature for the *in vivo* screening of synthetic pathways in which a single aldehyde is produced as an intermediate or end-product, and whose accumulation can be correlated to the catalytic efficiency of the upstream enzymatic reaction or can indicate a bottleneck in the downstream reaction in that pathway. This is nicely exemplified by our demonstration of glycoladehyde detection produced by the synthetic xylulose−1-P pathway.

Another key feature determining biosensor performance is its dynamic range of detection. We found that the sensitivity of our biosensor to aldehyde was in the range of 1–10 mM. However, in spite of this elevated minimum concentration, the application of the sensor should be possible when aiming at detecting aldehyde over-producing strains or enzymes. In addition, this relative poor sensitivity could be an advantage as it may avoid perturbation resulting from potential endogenous aldehydes, whose concentrations are actually relatively low due to the presence of many aldehyde reductases in *E. coli* (Rodriguez and Atsumi, 2014). Also, fluorescence saturation from the metabolite sensor at higher concentrations may constitute an advantage in metabolic engineering projects, in which high product titers are desired. Indeed, as a proof-of-principle application, we evaluated the feasibility of the system to monitor intracellular aldehyde production from a strain harboring the xylulose-1-phosphate pathway that can generate glycolaldehyde from xylose at a theoretical yield of 1 mol mol^−1^. A remarkable increase of the fluorescence signal dependent on xylose consumption was observed, thereby confirming the potential applicability of our high-throughput biosensor system toward metabolic and/or enzyme engineering projects upon minor optimization adjustments in each case scenario.

## Author contributions

CF and VM performed the experiments. JF and TW provided guidance for the experimental setups. CF, TW, and JF wrote the paper.

### Conflict of interest statement

The authors declare that the research was conducted in the absence of any commercial or financial relationships that could be construed as a potential conflict of interest.
